# Hooded seal *Cystophora cristata* foraging areas in the Northeast Atlantic Ocean—Investigated using three complementary methods

**DOI:** 10.1371/journal.pone.0187889

**Published:** 2017-12-06

**Authors:** Jade Vacquie-Garcia, Christian Lydersen, Martin Biuw, Tore Haug, Mike A. Fedak, Kit M. Kovacs

**Affiliations:** 1 Norwegian Polar Institute, Fram Centre, Tromsø, Norway; 2 Institute of Marine Research, Tromsø, Norway; 3 Scottish Oceans Institute, University of St Andrews, St Andrews, Scotland, United Kingdom; Centre National de la Recherche Scientifique, FRANCE

## Abstract

Identifying environmental characteristics that define the ecological niche of a species is essential to understanding how changes in physical conditions might affect its distribution and other aspects of its ecology. The present study used satellite relay data loggers (SRDLs) to study habitat use by Northeast Atlantic hooded seals (N = 20; 9 adult females, 3 adult males, and 8 juveniles). Three different methods were used in combination to achieve maximum insight regarding key foraging areas for hooded seals in this region, which have decline by 85% in recent decades: 1) first passage time (FPT); 2) vertical transit rate and; 3) change in dive drift rate. Generalized additive mixed models (GAMM) were applied to each method to determine whether specific habitat characteristics were associated with foraging. Separate models were run for the post-molting and the post-breeding seasons; sex and age classes were included in the GAMMs. All three methods highlighted a few common geographic areas as being important foraging zones; however, there were also some different areas identified by the different methods, which highlights the importance of using multiple indexes when analyzing tracking and diving data to study foraging behavior. Foraging occurred most commonly in relatively shallow areas with high Sea Surface Temperatures (SST), corresponding to continental shelf areas with Atlantic Water masses. All age and sex classes overlapped spatially to some extent, but the different age and sex groups showed differences in the bathymetry of their foraging areas as well as in their vertical use of the water column. When foraging, pups dove in the upper part of the water column in relatively deep areas. Adult females foraged relatively shallowly in deep water areas too, though in shallower areas than pups. Adult males foraged close to the bottom in shallower areas.

## Introduction

Identifying the environmental characteristics that define the ecological niche of animal species is essential to understanding how changes in physical conditions might affect their distribution, behavior, population dynamics and other aspects of their ecology. This task is particularly difficult to achieve in marine environments, which are difficult to observe directly. However, developments in biotelemetry have provided observational power that has dramatically increased our understanding of where and how marine animals use their habitats.

Foraging is a fundamental behavior that determines energy intakes and drives the evolution of an animal’s physiology and life history traits. Thus, foraging areas are vital areas to study in habitat use investigations. In recent decades, most studies of foraging behavior of marine predators have focused on surface tracks or diving records provided by biotelemetry/biologging instruments [1–8]. In the open ocean, these predators are assumed to use area-restricted search (ARS) behaviors when they encounter prey aggregations, reducing swimming speed and increasing the sinuosity of their tracks in areas where they find concentrations of food [9–10]. Thus, analyses of surface tracks, based on time spent in a given location [11], first passage time [12], or process-based models such as Switching State-Space Models or Hidden Markov Models [6, 13–14] are often used to analyze spatiotemporal patterns that are likely to be linked to foraging behavior. In parallel, when foraging at depth, these predators are generally assumed to reduce time spent transiting through the water column, spending more time at the bottom of their dives when they encounter prey [15]. Thus, different diving metrics such as bottom duration, wiggles or descent and ascent rates [16–18] are also frequently used to identify possible foraging areas. However, although these indirect methods are pertinent for inferring searching effort, they are not directly linked to foraging activity and resource acquisition and hence can be inaccurate or misleading regarding foraging success.

The recent development of other technologies such as esophageal and stomach temperature sensors [19], Hall sensors or accelerometers [20–23], as well as video cameras [24–25] are increasing our understanding of marine predator foraging activity by measuring prey capture attempts in the context of animal tracks. However, instrument recovery is required to collect most of these data-streams, and thus such instruments are not useful for many species in remote regions. An alternative method, based on body composition impacts on diving behavior has been developed to assess foraging success. This method monitors changes in the buoyancy of an animal through changes in measured dive characteristics, such as the descent or the ascent rates [26–28]. This approach gives useful information about changes in body condition and thus foraging performance for species that perform “drift dives”. During such dives, an animal drifts passively through the water column and their movements (up or down) are thus directly tied to their buoyancy [28].

Hooded seals (*Cystophora cristata*) are a deep-diving pinniped species that is distributed throughout the North Atlantic and adjacent Arctic marine areas [29–32]. They are a highly sexual dimorphic species; males and females weight 450 kg and 300 kg, respectively [33–34]. Hooded seals spend most of the year at sea, presumably foraging regularly outside the breeding and molting periods [29–32]. Two management stocks are recognized, although they cannot be distinguished genetically [35]. Hooded seals in the Northwest Atlantic (NW) stock breed in mid- to late March off the northern coast of Newfoundland (the Front), the Gulf of St Lawrence (the Gulf), and in Davis Strait [29, 36–37]. These animals migrate to southeastern Greenland by late June or early July to molt [36, 38]. Hooded seals in the Northeast Atlantic (NE) stock breed on the pack ice east of Greenland around Jan Mayen (West Ice) at the same time as NW seals [39]. They disperse broadly after breeding, but return to the pack ice east of Greenland in July to molt, usually just to the north of their breeding location [39–41].

Differences in migration patterns and diving behavior have been documented between the NW and the NE stocks [31, 37, 39, 42–44]. NW Atlantic seals follow regular round-trip migratory paths. The paths of males and females are spatially segregated for animals from the Front [43–44], but the paths of males and females from the Gulf overlap geographically [37]. NW Atlantic animals exhibit vertical segregation by sex during the post-breeding migration and during the post-molting season [37, 44]. Animals in the NE stock make unsynchronized, long excursions to sea following breeding and molting, returning intermittently to the ice east of Greenland [39]. During these excursions, pups and adults display vertical segregation in their diving behavior (pups dive shallower than adults do), despite a striking similarity in the overall spatial patterns of the two age groups [2]. Hooded seals from both stocks travel long distances during their annual cycles, diving almost continuously [30–31, 37, 39, 43–44]. The performance of drift dives has been documented for NW Atlantic hooded seals [45].

Habitat preference has been investigated for the NW population as a function of sex, age and season [43–44]. No such analyses have been conducted for the NE population, although conservation planning is particularly important for this stock at this time. Extreme declines, in excess of 85%, have occurred in the NE hooded seal stock over recent decades, resulting in the species as a whole shifting from Least Concern to Vulnerable on the IUCN Red List [46], and the listing of the NE stock as Endangered on the Norwegian Red List [47]. In this study, habitat preferences of the NE population of hooded seal were investigated to identify defining characteristics and the locations of their key foraging areas. Three different methods were used to achieve maximum insight: 1) first passage time (FPT); 2) vertical transit rates; and 3) change in drift rate. The potential roles of environmental variables, age, sex and season, in determining habitat use were explored using each method. Given that sea surface temperature and bathymetry were among the most important factors influencing habitat selection for the NW population [43–44], these variables, as well as ice concentration, were evaluated to determine their potential influences on foraging habitats of hooded seals in the NE population.

## Materials and methods

### Ethics statement

Animal-handling protocols were approved by the Norwegian Animal Research Authority (permit S-2007/1932-1) and the Governor of Svalbard.

### Deployment of devices and data collection

Twenty hooded seals (3 in July 2007–2 adult males and 1 pup—and 17 in March 2008–1 adult male, 9 adult females and 7 pups) were live-captured on the ice northwest of Jan Mayen Island (~73.86 N and 13.50 E) and instrumented with Conductivity-Temperature-Depth Satellite Relay Data Loggers (CTD-SRDLs) (Sea Mammal Research Unit, University of St Andrews). Adult animals were captured using nets, while the pups were hand-captured. The seals were weighed using Salter spring scales (± 0.5 kg) and sex was determined. Adult animals were immobilized with an intramuscular injection of Telazol® (1 mg kg^-1^ body mass for adult females; 0.75 mg kg^-1^ body mass for adult males). The CTD-SRDL tags were glued onto the hair on the back of the neck of adults and mid-dorsally on pups, using quick-setting epoxy.

The CTD-SRDLs collect and transmit—via the Argos satellite system (System Argos)—information on location, haul-out periods and diving behavior, as well as providing CTD up-casts on selected dives (for details; see [48–50]). Locations are estimated by the orbiting satellites and a location class (LC) is assigned to each position [48]. The full-resolution profiles of dives are compressed on-board, resulting in four at-depth points in addition to two surface points (start and end). A randomly selected subset of these compressed time-depth profiles are transmitted from each 6-hr period with the corresponding dive duration, maximum depth, and time spent at the surface following the dive as well as a selection of haul-out start and end times [48]. Additionally, one full CTD profile is transmitted from each 6-hr period, with 17 representative depth points, with corresponding temperature and conductivity values (for more details; see [50]).

### Data processing

All data processing and analyses were done using the R statistical framework [51]. Satellite-derived locations were first filtered using a speed, distance and angle filter (SDA filter; [52]) using the R package “argosfilter” [53]. This filter removes all LC Z values and points requiring unrealistic swimming speeds or unlikely turning angles [52]. The swimming speed threshold was set at 2 m/s and all spikes with angles smaller than 15 or 25 degrees were removed if their lengths were greater than 2.5 or 5 km, respectively [43–44, 52]. Then, locations were processed further using a Kalman filter under a state-space framework [54–55] using the R package “crawl” [56]. This filter incorporates a covariate for Argos location error when these are available (i.e. for location classes 0, 1, 2 and 3). In addition, a covariate encompassing the time the animal was hauled out was included, allowing movement along a track-line to stop during a haul-out event [54–55].

Filtered tracks were separated into the post–breeding season (encompassing the breeding period (March) until the beginning of the molting period (July)), and the post-molting season (from the time of molting until the beginning of the subsequent breeding period (defined herein as the first haul-out event after 15 February)). For adult animals tagged in the breeding period, the tags fell off 3–4 months after deployment, during the molt, while pups tagged in the breeding period retained their tags for about 14 months, at which time they underwent their first molt. For pups, the post-breeding season was defined as ending at the first haul-out event after June 15.

Filtered tracks were divided into trips. A trip was defined as an excursion starting from within 250 km of the mean deployment point (i.e. 73.86N and 13.50 E) in the drifting pack ice off the east coast of Greenland, to distant waters. If the animal returned to haulout on the ice within 250 km from the mean deployment point, it was defined as a complete trip. The mean deployment site was calculated as the average position of all tag deployments during the study, and preliminary analyses of the tracks showed that 250 km was a threshold that encompassed all returns of the adult animals (S1 Fig). Since pups do not molt (during their first year) or breed, they do not need to come back to the traditional areas where these activities take place. Thus, for pups, whatever travelling they performed was defined as ending/starting at the transition period between the temporal frames defined above for the different seasons, even if the movements took place outside the 250 km zone.

Dives were analyzed based on the time-depth inflection points provided by the CTD-SRDL tags as well as their start and end points. Descent and ascent rates (m/s) were calculated for each dive as the ratio between the difference in depth and the difference in time between the start point and the first time-depth point of the dive and between the last time-depth point and the end point of the dive, respectively. Locations of dives were estimated by linear interpolation along the filtered tracks. Each dive was also assigned to a season and given a trip number according to their time stamps.

### Movement and diving parameters

Three movement parameters and three diving parameters were compared between age/sex classes and seasons. The three movement parameters were calculated for each trip while diving parameters were calculated for each dive. The three movement parameters were the overall azimuth (°) (i.e. angle between the straight line defined by the first point of the trip and the most distant point and the North-South axis), the total duration of the trip (min) and the maximum distance (km) measured between the beginning of the trip and the most distant point. The overall azimuth was included in the analyses of all trips. The other movement parameters were only considered for complete trips. The three diving parameters were maximum dive depth (m), dive duration (s) and post-dive surface duration (s), which were extracted from CTD-SRDLs data directly.

A Rayleigh test was used to test whether the directionality of the trips were random. Then, the azimuth was transformed to its cosine and sine and the potential influences of age/sex classes and season were tested on both orientation parameters (i.e. on the North/South and the East/West axis) separately. Because data were not available for all age/sex classes for all seasons, approaches involving linear models were considered unsuitable. The comparisons of each movement and diving parameter between the different age/sex classes, within each season, and between the same age/sex classes in different seasons, were thus investigated using Wilcoxon-Mann-Whitney tests.

### Environmental data extraction

Three environmental variables were calculated for each dive based on their locations and time-stamps. Bathymetry (i.e. water depth) was extracted from the 0.01-degree resolution ETOPO 1 Arc-Minute global relief data set from the National Geophysical Data Center, NOAA [57]. Sea ice concentrations were extracted from the 25x25 km resolution data set Nimbus-7 SMMR and DMSP SSM/I-SSMIS Passive Microwave Data, Version 1 from the National Snow and Ice Data Center [58]. Sea surface temperatures (SST) were estimated for each dive location using the temperature corresponding to the shallowest data point within a dive (i.e. 6 m—collected by the CTD-SRDLs). Because CTD profiles were not available for every dive, an interpolation method was used, when necessary, to assign a SST value to each dive. Daily averages of each environmental variable were used to overcome the problem of incompatible scales of resolution of the different data sets and the potential inaccuracy of the location estimates for each dive. A daily mean maximum diving depth was also calculated to provide general information about where in the water column each animal was found on a given day.

### Foraging indexes

#### First passage time

FPT is defined as the time required for an animal to cross a circle of a given radius [12, 59]. If an animal travels at a slow rate or performs a lot of turns, it will take longer to cross the circle than if the animal travels at a faster rate or in a straighter line. Thus, FPT provides a proxy for horizontal search effort. Here, FPT was calculated following [12], using the ‘adehabitat’ R-package (version 1.8–3; [60]). Because location data was sampled independently of speed along the track, a larger number of location points will be sampled in areas of low speed compared to areas of high speed, which will create a sampling bias. Thus, locations were interpolated such that they were regularly spaced at 5 km intervals along the track-line [12]. The scale at which animals focused their search effort (i.e. area restricted search ARS) was then identified by maximizing the FPT variance, testing different radii (for more details see [12]) and finally the FPT values corresponding to the identified scale were calculated for each location [12, 59, 61]. The spatial scale of foraging was initially investigated by age/sex class and season and subsequently all trips were used to identify a common ARS scale. In accordance with previous studies on hooded seals, radii varying between 5 km and 100 km were tested in 1 km steps [43–44]. FPT values related to haul-out events (i.e. distance to a haulout shorter than the scale identified) were removed to avoid bias. Finally, FPT values were averaged for each day to coincide with the temporal scale of the environmental variables.

#### Vertical transit rates

Ascent and descent rates of dives contain information that can be used to estimate foraging activity in a large number of marine predators [62–65]. Generally, animals increase their ascent and decent rates when they have found food, in order to spend as much time as possible feeding (at the bottom of dives) [65]. By combining these two parameters for each dive using a Principal Components Analysis (PCA), one obtains an index of “foraging intensity” [66]. Since air in the lungs can have a large influence on an animal’s buoyancy during shallow diving (i.e. <100 m; [28, 67]), ascent and descent rates were weighted according to the mean depth at which the descent ended and ascent began. Foraging indexes associated with a mean transit depth of 100 m (or deeper) were given the value 1, while foraging indexes associated with a shallower mean transit depth were given a value from 0 at 0 m, up to 1 at 100 m in a non-linear fashion as described in [28]. These foraging indexes were then averaged within each day to be comparable with other foraging indexes and environmental variables.

#### Change in drift rates

Drift dives are dives where animals drift passively through the water column for a substantial portion of the dive. These dives are thought to be resting/sleeping or food processing dives [26], and since the vertical rate of change in depth during the drift phase varies depending on the seal’s buoyancy (i.e. body composition), they can be used as indicators of foraging success by calculating the daily change in vertical drift rates over time (see [28] for more details). In the present study, drift dives were identified using a semi-supervised classification method [16] based on a tree-based random forest algorithm [68]. Drift segments were identified and drift rates were calculated as described in [28]. Since positive drift segments are rare, but have the potential to impact the results heavily, visual validations of these events were conducted before including them in the analyses [28]. For each trip, a smoothed time series of drift rates was constructed using a weighted, constrained, beta spline [69–70]. Only trips with one or more identified drift segment per 2 days were included in this analysis. The same weighting scheme as for transit rates was applied to drift rates entered into the beta splines. To minimize the influence of erroneous drift rates associated with dives that had a low probability of being true drift dives, weights based on the depth were multiplied by weights based on the classification probability from the random forest algorithm. Daily drift rates were then predicted from the fitted spline along each trip, and finally a daily change in drift rate was calculated as the first derivative of the daily-predicted drift rates, to coincide with other foraging indexes and environmental variables.

### Modelling approach

The three daily foraging indexes were investigated separately in relation to bathymetry and SST. Since sea ice was largely restricted to the area that marked the start and end points of offshore pelagic trips (in ice-free waters), this environmental variable was not included in these analyses. Generalized additive mixed models (GAMM; ‘uGamm’ function in the R package ‘MuMIn’) were used to explore relationships. To simplify analyses, the models were fitted by season. Age/sex classes were taken into account in each of the seasons. For each type of model, explanatory variables were first standardized [71] after verifying that metrics were not highly correlated (≤ 0.8). Individual IDs, as well as trip number, were included in the models as random effects. All the combinations of metrics were tested for each type of model and the models were ranked according to the Akaike’s information criterion, AIC [72]. Models having ΔAIC < 2 were considered to be candidate models in this study [72]. However, in the case where candidate models differed by only 1 parameter, the candidate model with the lowest number of parameters was selected [72]. An adjusted R^2^ was extracted for each selected GAMM.

### Diving behavior when foraging

Areas with daily FPT values higher than the 75% quantile were considered to be important foraging areas. Within these zones, the mean maximum depth per day as well as the ratio between the mean maximum depth and the bathymetry for the different age/sex classes within each season, and between the same age/sex classes for different seasons, were compared using Wilcoxon-Mann-Whitney tests.

## Results

### Movement and diving parameters

**Fig 1:**
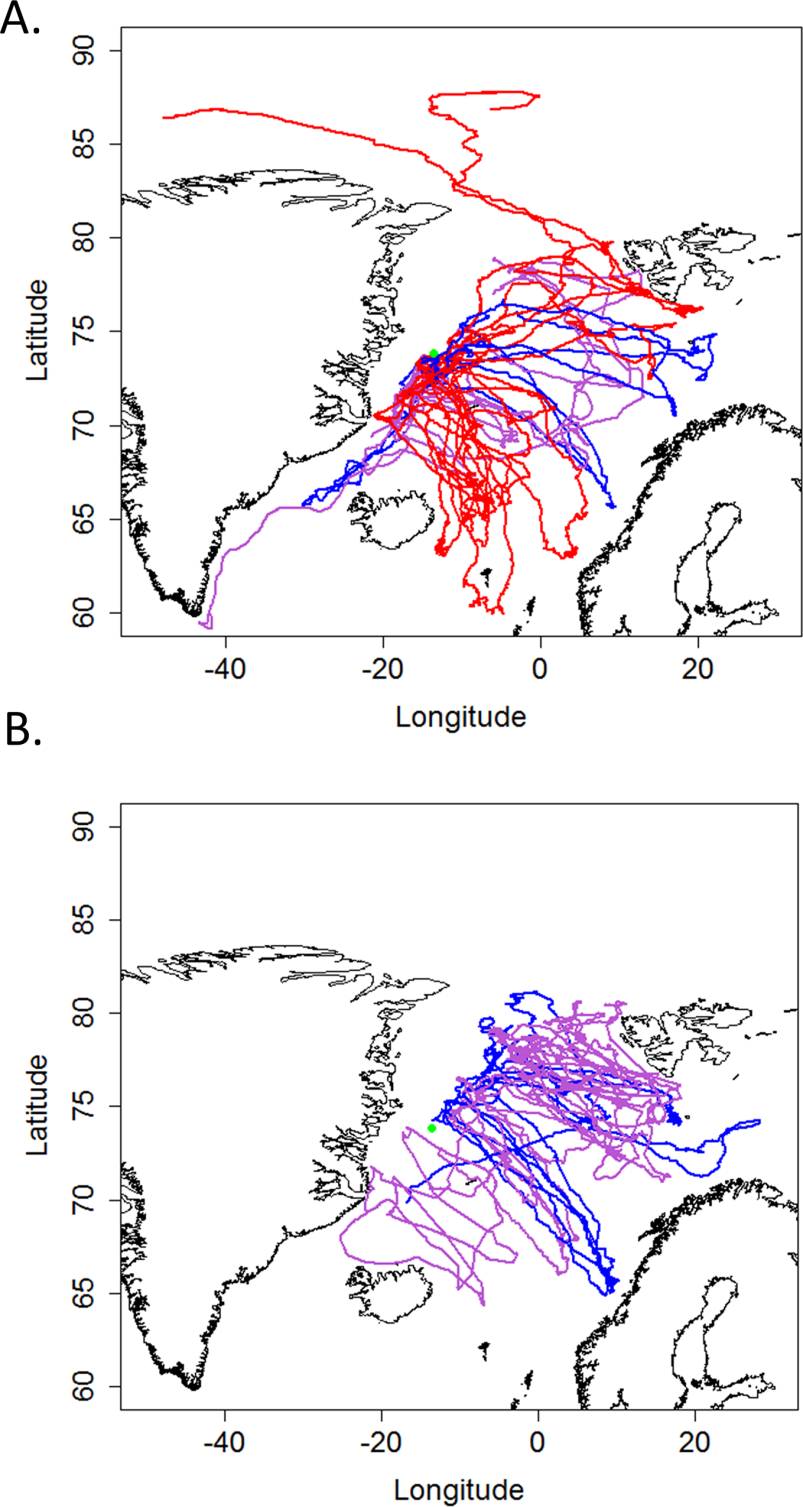
**Hooded seal foraging trips during A) the post-breeding season and B) the post-molting season**. Blue lines represent adult males, red lines represent adult females and purple lines represent the pups. The green dot represents the mean deployment point.

Tags on two of the 20 instrumented animals provided data for a short period of time (1 day and 25 days, respectively, for 2 pups). These individuals were therefore removed from further analyses. The remaining 18 seals undertook a total of 45 foraging trips (i.e. 2.50 ± 1.8 trips per animal) of which 27 were complete round-trips, over large areas spanning from East Greenland to the Norwegian coast (Fig 1). The average duration of complete trips was 67 ± 42 days (range 13–214 days) and the average maximum distance was 859 ± 347 km (range 248–1,588 km). One male conducted five complete round-trips between similar start and end points (S2 Fig). Each of the trips made by this individual lasted on average 50 ± 6 days (range 42–55 days) and covered an average maximum distance of 1,028 ± 383 km (range 457–1,273 km).

The directionality of trips was not random (Rayleigh test, 0.61, p-value < 0.001). Most of the seals migrated in a southeasterly direction from their departure point (Fig 1 and Table 1). No difference was found between the different age/sex classes or seasons with regards to trip orientation (i.e. cos(azimuth) and sin(azimuth), Table 1), except that males and pups displayed different headings in the post-molting period (p-value = 0.01), with the males travelling in a more easterly direction than the pups. Significant differences were detected between the various age/sex classes and seasons in the maximum distance of trips. The pups in the post-breeding season undertook shorter trips than pups in the post-molting season (p-value = 0.01; Table 1). In addition, pups in the post-breeding season undertook shorter trips than adults of both sexes (p-value = 0.003 for pups vs females and p-value = 0.036 for pups vs males; Table 1). Correspondingly, the duration of the trips made by pups in the post-breeding period was significantly shorter than for pups in the post-molting season, and also shorter than for females in the post-breeding season (Fig 1 and Table 1; p-value = 0.048 between pups in post-molting vs post-breeding; p-value = 0.045 between pups vs females in post-breeding).

**Table 1:** Movement parameters for hooded seal adult males, adult females and pups during the post-breeding and the post-molting seasons. Med. = median, and Q (25%) and Q (75%) = the 25% and 75% quantiles, respectively. The number of trips per age/sex class and season is detailed in parenthesis. These trips were conducted by 3 males, 9 females and 6 pups in the post-breeding period and by 2 males and 5 pups in the post-molting period. The second number in parenthesis represents the number of complete trips.

		MOVEMENT PARAMETERS											
		Cos(azimuth) (NS)			Sin(azimuth) (WE)			Trip distance (km)			Trip duration (days)		
Seasons	Classes	Med	Q(25%)	Q(75%)	Med	Q(25%)	Q(75%)	Med	Q(25%)	Q(75%)	Med	Q(25%)	Q(75%)
**Post-breeding**	**Pups (N = 11 (5))**	-0.61	-0.81	-0.07	0.58	-0.13	0.85	509.65	289.77	528.66	32.25	28.88	42.37
**Post-breeding**	**Females (N = 12 (8))**	-0.77	-0.93	0.50	0.47	0.29	0.70	1059.35	855.02	1234.79	63.44	47.73	89.92
**Post-breeding**	**Males (N = 5 (3))**	-0.36	-0.55	-0.01	0.90	0.57	0.93	1155.41	930.22	1159.07	47.48	44.99	69.41
**Post-molting**	**Pups (N = 11 (7))**	-0.81	-0.85	-0.68	0.57	0.34	0.67	733.51	651.98	928.04	69.17	41.32	102.09
**Post-molting**	**Males (N = 6 (4))**	-0.61	-0.69	0.03	0.79	0.72	0.93	1071.42	806.42	1233.22	54.07	51.86	78.11

A total of 73,669 dives were included in the analyses. The number of dives reported for pups was on average 5,018 ± 2,343, while adult males and adult females reported 6,046 ± 3,534 and 2,824 ± 654 dives on average, respectively. The overall average dive depth for all animals was 173 ± 131 m (range 10–1,149 m) and the average dive duration was 9 ± 6 min (range 1–87 min). Adult males (N = 18,139) dove to 265 ± 157 m (range 10–1,149 m) and to 170 ± 125 m (range 10–1,084 m) for average durations of 14 ± 6 min (range 1–66 min) and 12 ± 8 min (range 1–87 min) during the post-breeding (N = 6,725) and the post-molting (N = 11,414) seasons, respectively. Adult females (N = 25,420) dove to 245 ± 122 m (range 10–1,034 m) for an average duration of 12 ± 5 min (range 1–53 min) in the post-breeding season, while pups (N = 30,110) dove to 105 ± 83 m (range 10–644 m) and to 88 ± 64 m (range 10–441 m) for average durations of 5 ± 3 min (range 1–32 min) and 5 ± 3 min (range 1–24 min) during the post-breeding (N = 8,851) and the post-molting seasons (N = 21,259), respectively. Pup dives were significantly shallower and of shorter duration than the diving of adults and their surface durations were shorter (p-value < 0.001; Table 2). Males dove deeper and longer than females in the post-breeding season and they spent less time at the surface (p-value < 0.001) (Table 2). Generally, the seals dove shallower, with shorter durations and shorter surface times, in the post-molting season compared to the post-breeding season (p-value < 0.001) (see Table 2 for details).

**Table 2:** Diving parameters for hooded seal adult males, adult females and pups during the post-breeding and the post-molting seasons. Med. = median, and Q(25%) and Q(75%) = the 25% and 75% quantiles, respectively. The number of dives per age/sex class and season is detailed in parenthesis. These dives were conducted by 3 males, 9 females and 6 pups in the post-breeding period and by 2 males and 5 pups in the post-molting period.

		DIVING PARAMETERS								
		Depth (m)			Duration (min)			Surface duration (min)		
Seasons	Classes	Med	Q(25%)	Q(75%)	Med	Q(25%)	Q(75%)	Med	Q(25%)	Q(75%)
**Post-breeding**	**Pups (N = 8,851)**	75.00	40.00	152.50	4.00	2.50	8.00	1.02	0.85	1.35
**Post-breeding**	**Females (N = 25,420)**	231.30	157.50	331.30	12.00	9.00	15.00	1.68	1.35	2.07
**Post-breeding**	**Males (N = 6,725)**	251.30	150.00	346.30	14.00	10.50	17.00	1.60	1.35	1.93
**Post-molting**	**Pups (N = 21,259)**	70.00	45.00	112.50	4.50	3.00	6.50	1.07	0.77	1.18
**Post-molting**	**Males (N = 11,414)**	132.50	80.00	236.30	12.00	6.50	17.50	1.51	1.27	1.85

### Foraging indexes

#### First passage time

**Fig 2:**
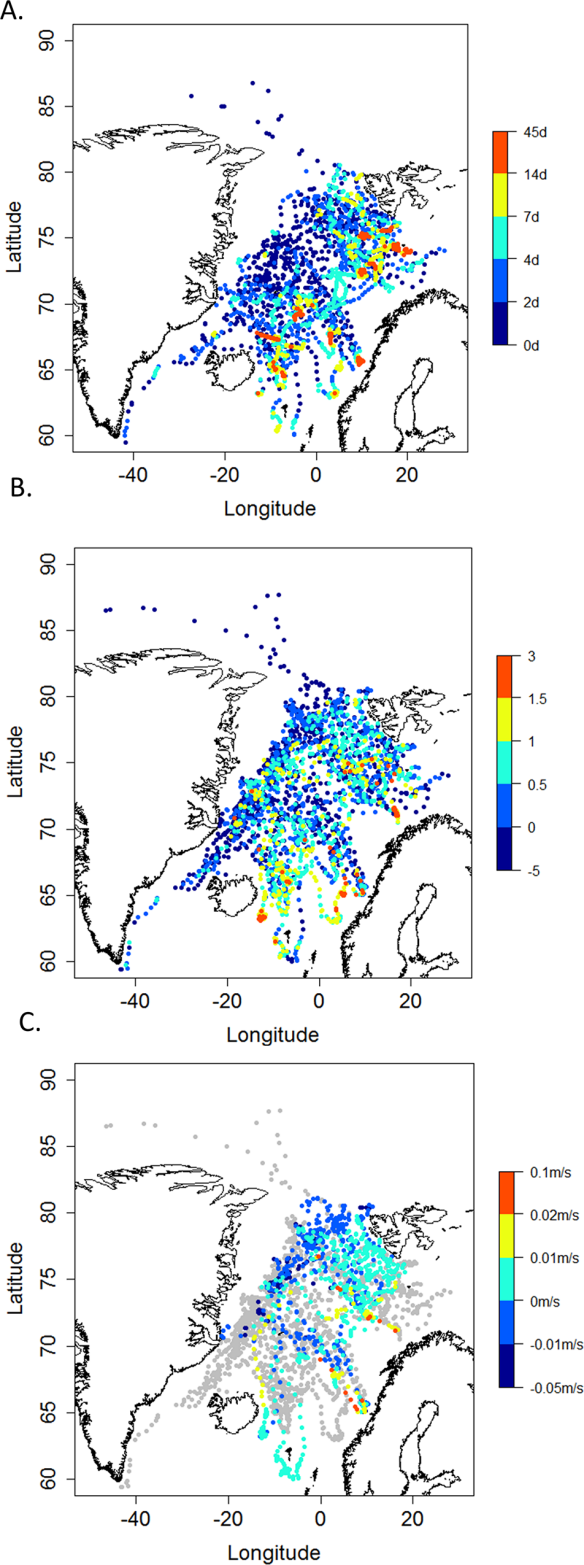
**Hooded seal foraging index per day using A) first passage time (days), B), PCA-derived foraging intensity (based on combined vertical transit rates when diving (no unit)), and C) the change in drift rate (m/s).** First passage time was calculated using a 55 km scale. For each graph—the warmer the colors, the higher the values.

The FPT analysis revealed a lot of variability in the scale at which the hooded seals focused their ARS activity (Table 3). A radius of 55 km was chosen for the FPT analysis in order to facilitate comparisons between age/sex classes and seasons (Table 3). The daily FPT values at this scale for all classes combined ranged from 1 to 44 hr. Pups ranged from 1 to 44 hr, while adult males ranged from 1 to 33 hr. Adult females ranged from 1 to 16 hr. The areas with high FPT values were generally found close to the most distant reaches of trips. These locations occurred near the east coast of Iceland, around the Faeroe Islands and along the shelf break between Bjørnøya and the Norwegian mainland, as well as along the coast of the Norwegian mainland (Fig 2A).

**Table 3:** Area restricted search (ARS) scale for each age/sex class per season.

Classes	Seasons	Number of trips	Radius of the ARS (km)
Pups	Post-breeding	11	5
Females	Post-breeding	12	80
Males	Post-breeding	5	25
Pups	Post-molting	11	40
Females	Post-molting	0	NA
Males	Post-molting	6	55
All classes	All seasons	45	55

#### Vertical transit rates

The first axis of the PCA, performed on the combined ascent and descent rates, explained 74% of the total variance in the data, and was therefore used independently as an index of foraging intensity. The daily average transit rate ranged from -3.06 to 2.43 for pups, from -2.31 to 2.09 for adult males and from -2.44 to 1.89 for adult females. Areas classified by this method as preferred foraging areas (high ascent and descent rates) overlapped with areas identified by the FPT method (i.e. near the east coast of Iceland and along the shelf break between Bjørnøya and the Norwegian mainland): However, the transit rate analysis also identified foraging activity dispersed along the tracks of the animals (Fig 2B).

#### Change in drift rates

A total of 2,040 dives contained drift segments that fit the selection criteria; 1,431 (70%) of these were performed by pups. The number of drift dives extracted per trip was 45 ± 60 (ranging from 0 to 265), depending on trip duration and individual variability in performance of drift diving. Most of the drift dives extracted (2,039 dives) showed a negative slope during the drift segment; only one dive (for a pup) showed a positive slope during a drift; after visual inspection, the drift dive for this pup was excluded from the analyses. The mean maximum drift dive depth across all trips was 245 ± 125 m and the mean drift dive duration was 14.7 ± 6.4 min. Adult males and adult females drifted at average rates of -0.31 ± 0.10 m/s (range -1.09 –-0.02) and -0.33 ± 0.08 m/s (range -0.52 –-0.03), respectively, while pups drifted at an average rate of -0.25 ± 0.07 m/s (range -0.56 –-0.023). Only 19 of 45 trips, performed by 9 individuals (i.e. 5 pups, 1 male and 3 females), were used to predict daily drift rates with the weighted constrained beta splines because the remaining trips did not contain enough drift dives to enable detection of variation in condition of the seals along trips (S3 Fig). The daily change in drift rate values ranged from -0.05 to 0.08 m/s overall; -0.05 to 0.02 m/s for pups, -0.02 to 0.08 m/s for adult males and -0.01 to 0.01m/s for adult females. Areas where the seals experienced positive changes in drift rates (i.e. improved body condition) generally overlapped with the areas identified as important foraging areas by the other methods; they occurred along the east coast of Iceland and along the shelf break between Bjørnøya and the Norwegian mainland. However, some other favorable foraging areas were detected by this method, such as the southwest coast of Svalbard (Fig 2C).

### Habitat preferences

**Fig 3:**
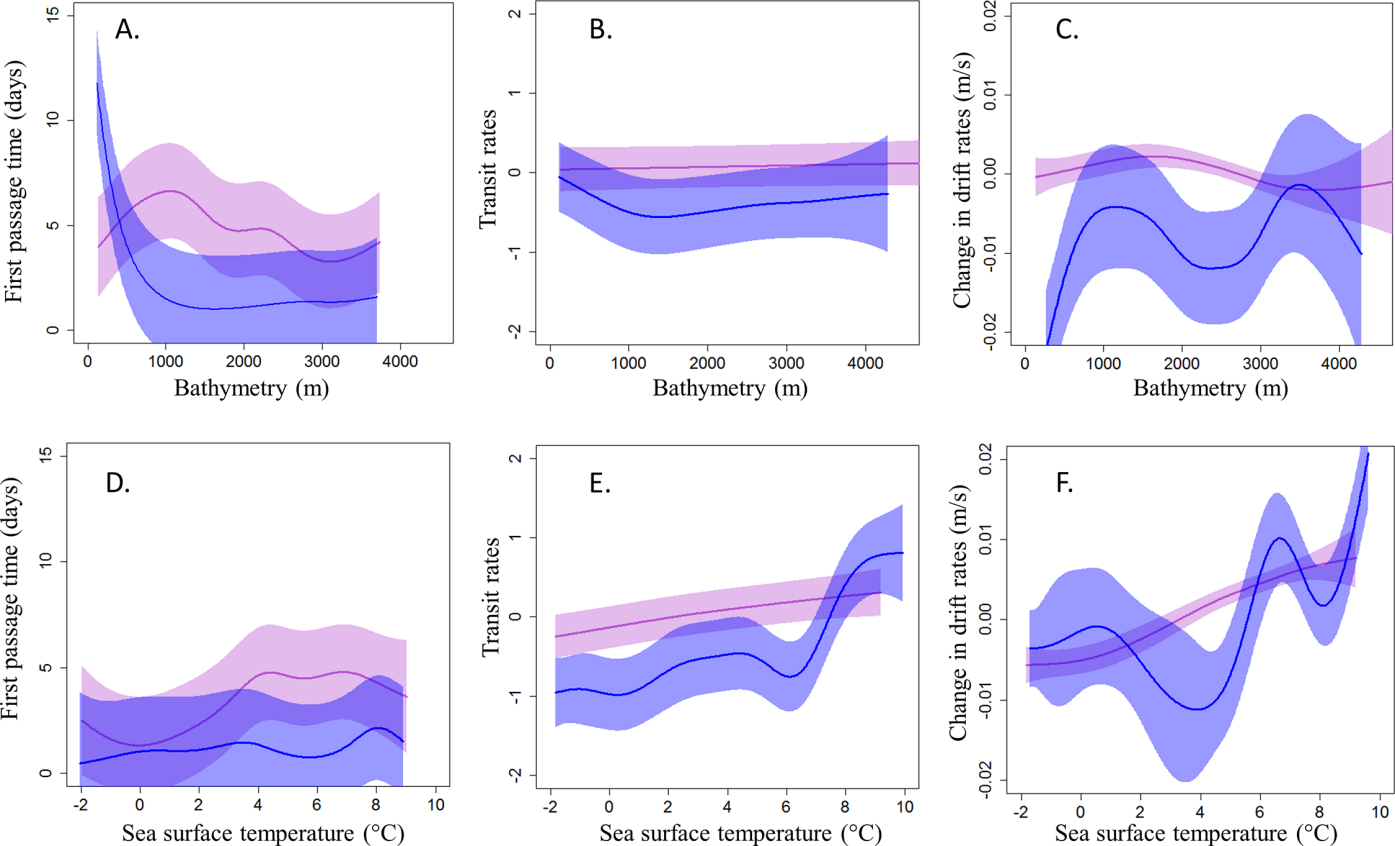
**Effect of bathymetry and SST on foraging indexes of hooded seals using (A) first passage time (days), (B) vertical transit rates when diving (PCA derived combination of ascent and descent rates, no unit), and (C) change in drift rates (m/s), for pups (purple) and adult male (blue) hooded seals during the post-molting season.** Fitted estimates from best models (solid curves) are represented along with the CIs (polygons) calculated from the variance–covariance matrices of the random effects of the fitted models.

**Fig 4:**
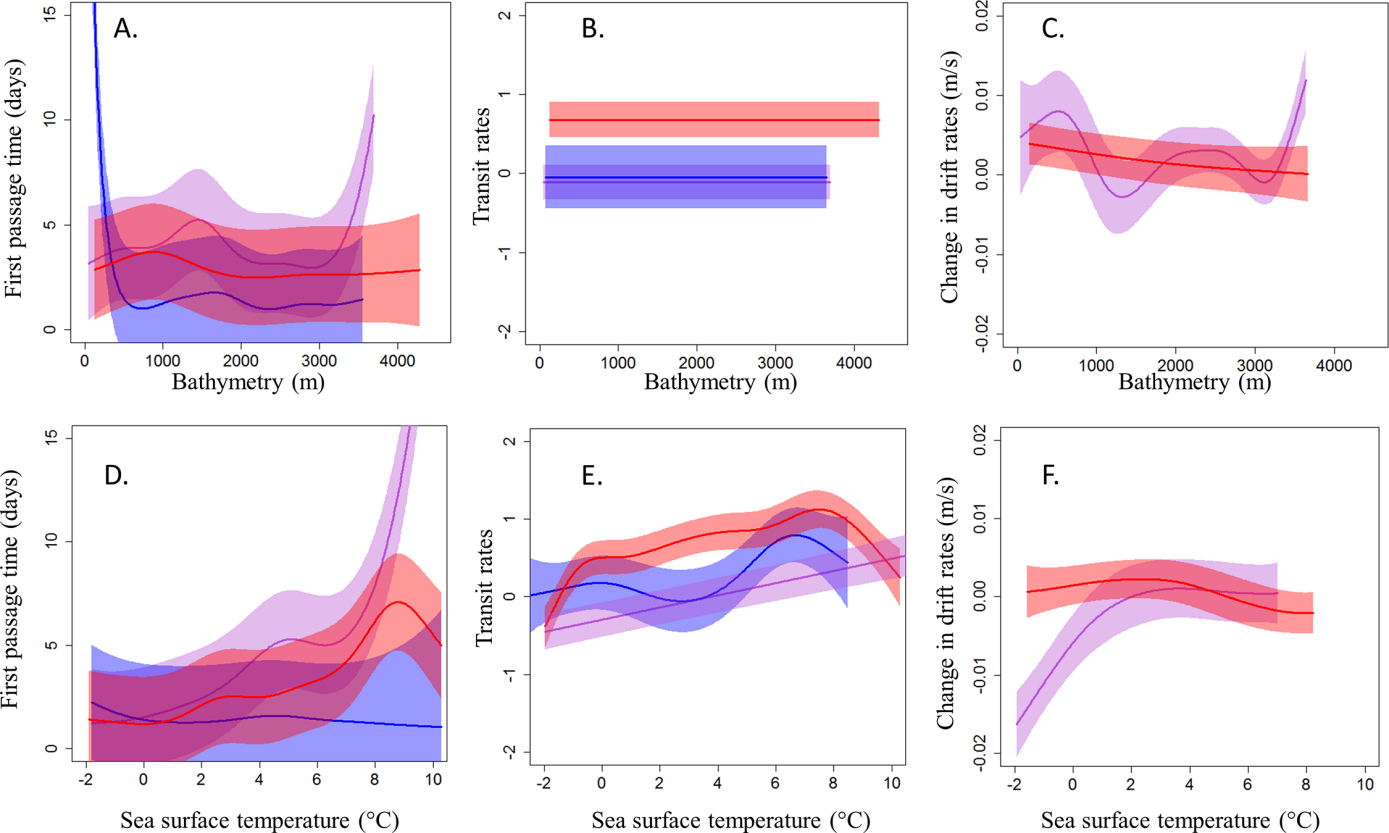
**Effect of bathymetry and SST on foraging indexes of hooded seals using (A) first passage time (days), (B) vertical transit rates when diving PCA derived combination of ascent and descent rates, no unit), and (C) change in drift rates (m/s), for pups (purple), adult females (red) and adult males (blue) during the post-breeding season.** Fitted estimates from the best models (solid curves) are represented along with the CIs (polygons) calculated from the variance–covariance matrices of the random effect of the fitted models.

Along their tracks, the hooded seals crossed areas characterized by daily average bathymetry values from 47 m to 5,144 m (1,853 ± 1053 m) and by daily average SSTs ranging from -1.9°C to 10.7°C (2.9 ± 3.4°C). All selected best models, for each index of foraging and each season, included both SST and bathymetry (Table 4, Figs 3 and 4), except for the model investigating the vertical transit rates during the post-breeding season, which included only the SST (Table 4, Figs 3 and 4).

**Table 4:** Hooded seal habitat preferences identified using GAMMs. Bathymetry (Bathy), sea surface temperature (SST) and the age/sex classes (i.e. Pups, Females and Males) were explored.

Foraging indexes	Seasons	Best models	df	AIC	Adjusted R2
First passage time	Post-molting	1+s(Bathy,by = classes)+s(SST,by = classes)+classes	13	1956.4	0.44
Vertical transit rates	Post-molting	1+s(Bathy,by = classes)+s(SST,by = classes)+classes	13	2213.1	0.12
Change in drift rates	Post-molting	1+s(Bathy,by = classes)+s(SST,by = classes)	13	-4660.1	0.37
First passage time	Post-breeding	1+s(Bathy,by = classes)+s(SST,by = classes)+classes	18	1857.7	0.45
Vertical transit rates	Post-breeding	1+s(SST,by = classes)+classes	12	2326	0.35
Change in drift rates	Post-breeding	1+s(Bathy,by = classes)+s(SST,by = classes)	12	-2836.5	0.47

Bathymetry was an important variable for all age and sex groups (Figs 3 and 4) with regard to foraging habitat selection. During the post-molting period, pups spent more time in areas with intermediate water depths (1,000–2,000 m, Fig 3A) and it was in these areas that they improved their body condition (Fig 3C). Adult males selected areas with shallower water depths than pups (0–500 m, Fig 3A) and they improved their body condition at these sites (Fig 3C). Body condition of adult males also improved at greater water depths, in areas where they did not spend much time (Fig 3C). However, the vertical transit rates of pups and adult males were not affected greatly by bathymetry (Fig 3B). During the post-breeding season, pups selected areas with intermediate water depths (1,000–2,000 m) but also spent time in much deeper areas (3,000 m) (Fig 4A). Pups improved their body condition in areas with intermediate and greater water depths, as well as in quite shallow areas in the case of some individuals, in places where they spent only average amounts of time (Fig 4C). Adults of both sexes selected somewhat shallower water depths than pups; males selected shallower areas than females (0–500 m for males vs 500–1,500 m for females, Fig 4A). Females improved their body condition at the water depths where they spent most time (Fig 4C). Vertical transit rates were not affected by bathymetry for any age/sex class in the post-breeding season, similar to the post-molting season. (Fig 4B).

SST was important for habitat selection for all age and sex classes (Figs 3 and 4). During the post-molting season, pups selected areas with SSTs ranging from 4–8°C and increased their vertical transit rates (Fig 3D and 3E) and improved their body condition in these areas (Fig 3F). Adult males selected areas with a narrower range of SSTs during the post-molting season compared to pups (7–8°C, Fig 3D and 3E), and their body condition improved markedly (Fig 3F). During the post-breeding season, pups again selected areas with SST between 4–8°C, increased their vertical transit rates in these areas (Fig 4D and 4E), and improved their body condition (Fig 4F). Both adult females and males selected areas with narrower SST ranges (7–8°C, Fig 4D and 4E) than the pups. SST showed no relationship with FPT among males (Fig 4D). The body condition of females improved in areas with relatively cold water (Fig 4F).

### Diving behavior when foraging

The areas with daily FPT values higher than the 75% quantile (i.e. 6.51 days) were in many cases identified as favorable foraging areas using the vertical transit rates and the change in drift rate methods. In these areas, pups dove to significantly shallower depths than adult animals (p-value < 0.001 for any comparison between pups and adults, except between pups and males in the post-breeding period (with p-value = 0.04; Table 5)). Pups dove in the upper parts of the water column when foraging while adult females dove somewhat deeper in the water column, though they remained pelagic (p-value < 0.001 for any comparison; Table 5). Adult males foraged close to the seafloor (p-value < 0.001 for any comparison; Table 5), though males’ foraging dives were shallower than those of females because they occupied shallower areas during the post-breeding season (p-value < 0.001; Table 5, also see Fig 4A). Males dove closer to the bottom during the post-breeding season compared to the post-molting season (p-value < 0.01; Table 5). Pups dove deeper during the post-breeding season than during the post-molting season when in foraging areas, but pups dove closer to the bottom during the post-molting period compared to the post-breeding period (p-value 0.03 and p-value = 0.02 respectively; Table 5).

**Table 5:** Hooded seal diving behavior when foraging for adult males, adult females and pups during the post-breeding and the post-molting seasons. Med. = median, and Q(25%) and Q(75%) = the 25% and 75% quantiles, respectively. The number of days per age/sex class and season is detailed in parentheses. These records represent diving activities conducted by 2 males, 6 females and 5 pups in the post-breeding period and by 2 males and 5 pups in the post-molting period.

		DIVING BEHAVIOR WHEN FORAGING					
		Depth (m)			Depth/Bathymetry		
Seasons	Classes	Med	Q(25%)	Q(75%)	Med	Q(25%)	Q(75%)
**Post-breeding**	**Pups (N = 75)**	155.65	69.28	196.54	0.05	0.03	0.07
**Post-breeding**	**Females (N = 107)**	303.91	267.21	337.25	0.25	0.16	0.35
**Post-breeding**	**Males (N = 27)**	168.13	108.43	221.12	0.91	0.86	0.97
**Post-molting**	**Pups (N = 178)**	106.92	76.80	142.89	0.06	0.04	0.09
**Post-molting**	**Males (N = 72)**	161.50	134.06	228.61	0.72	0.51	0.81

## Discussion

### Age and sexual segregation

This study supports earlier findings that hooded seals are long-distance swimmers that occupy vast oceanic areas [39, 43]. In the present study, hooded seals travelled between the east coast of Greenland and the west-coast of the Norwegian mainland, and they visited areas from south of the Faroe Islands north almost to the North Pole (88,5°N). No seasonal movement patterns were identified with regard to the directionality of trips. These results support conclusions from [39] stating that hooded seals from the NE population do not display any set seasonal migration patterns; they occupy ice-covered waters off the east coast of Greenland for breeding and molting, and make long trips to distant waters to feed. This behavior differs from that of NW hooded seals, which undertake a more regular round-trip migratory pattern. Such a difference likely results in different constraints on animals in the two stocks as to where they find food. The trips documented in the present study were mainly carried out in a southeasterly direction, although some animals moved southwest or northwest of the breeding/molting areas. Two adult females almost reached the North Pole before their tags were dropped during their molt; subsequently the tags drifted south passively with the ice (until the batteries ran out or the tags sank). It is interesting to note that despite severe ice reductions within the last decades, the movements of hooded seals from the NE population seem to be very similar to the patterns reported in the early 1990s [39]. Most trips in the present study lasted between one and three months and the animals travelled distances between 250 km and 1,600 km. During these trips, animals performed different diving activities depending on the season. These results are also consistent with earlier findings [31].

The activity budgets of many marine mammal species differ markedly between the sexes [e.g. 73–76]. Spatial distribution, diving behavior and diet can vary between males and females, with the two sexes often using different foraging tactics [77–79]. These differences are usually linked with body size dimorphism. Hooded seals are highly dimorphic animals, which leads us to expect spatial segregation (in two or three dimensions) between foraging areas for males and females. Earlier studies of NW Atlantic hooded seals found both geographical and vertical segregation between the sexes, except for animals breeding in the Gulf of St Lawrence; for this subpopulation males and females overlap spatially throughout the year [37, 43–44]. In the present study, no difference were found between males and females for movement parameters (trip distance, trip duration, cos(azimuth) and sin(azimuth)) (Table 1), which is in agreement with the findings of [39] for the NE population. However, in contrast to the findings of [31], this study found that males and females dove differently; males generally dove deeper and longer while travelling, with less time at the surface compared to females.

Hooded seal pups had similar distribution patterns to the adults in this study. However, during the post-breeding season, the maximum distances travelled by pups as well as their trip durations were shorter, and their dives were shallower with shorter surface times between them compared to adult animals. These results are consistent with the fact that pups are likely to be more physiologically constrained than adults [80]. In pinnipeds, pups undergo a period of physiological and behavioral development of dive skills [81]. In contrast to some other seal species, such as northern (*Mirounga angustirostris)* and southern elephant seals (*Mirounga leonina)*, that develop diving skills prior to dispersal from natal areas, while they are still being fed by their mothers [82–83], hooded seal pups are weaned when they are only a few days old and must learn to swim and dive on their own [42, 84]. Although hooded seals are born with fully developed hemoglobin stores, their myoglobin levels are only 25–30% of adults’ levels [85–86], which means that pups rely heavily on anaerobic metabolism during early diving activities [87]. Their myoglobin levels increase concomitantly with increased swimming activity [85–86], which is consistent with the previously reported rapid development of diving capacity in wild [42] and with the results of this study that showed progression in their diving and swimming skills from the post-breeding period to the post-molting period.

### Foraging indexes

In the present study, three different methods were used to infer foraging based on animal tracks and diving records. All of these methods showed that hooded seals forage all along their track lines to some degree. In addition, they all highlighted a few key foraging area where concentrated foraging took place, including at the shelf break between Bjørnøya and the Norwegian mainland and on the east coast of Iceland.

The different methods used in this study to explore hooded seal foraging also displayed some differences. The FPT method showed that hooded seals display easily identifiable areas where they perform ARS behavior, which is consistent with findings from the NW population [43]. Important areas (high FTPs) were found mainly at the most distant parts of the trips, along the shelf break between Bjørnøya and the Norwegian mainland and along the coast of Iceland. Newly independent pups in the post-breeding season did not perform identifiable ARS behavior. This is not surprising given that a period of exploration (with no parental guidance) must likely take place before consistent foraging patterns develop. Despite identification of a few common key foraging areas with the FPT method, the vertical transit rates method showed that hooded seals increased their vertical search effort at numerous sites along their paths. This result is surprising given the normal assumption that animals have two distinct behavioral patterns (active searching for food vs transit) during foraging trips [9]. However, this result is consistent with findings for southern elephant seals [23, 65, 88], which attempt to capture prey quite continuously along their paths. Although, like hooded seals, elephant seals do feed intensively in some specific areas [65]. Both the FPT analyses and the vertical rate change analyses suggest that hooded seals are opportunistic foragers that feed along their paths, probably in response to prey densities. A substantial amount of pelagic, planktivorous schooling fish could be eaten by hooded seals along their transit routes, especially herrings (*Clupea harengus)*, blue whiting (*Micromesistius poutassou)* and Atlantic mackerel (*Scomber scombrus)*. These fish species spend most of the summer and autumn feeding in the upper water layers in areas traversed by hooded seals [89–91].

Finally, the change in drift rate method highlighted the shelf break between Bjørnøya and the Norwegian mainland, as well as the coast of Iceland, as particularly important areas for fattening. This suggestion is consistent with the main areas identified as having high FPTs, indicating that successful foraging is taking place at these sites; energy intake is higher than energy consumption in these areas. However, some additional favorable foraging zones were detected by this method, such as the southwest coast of Svalbard. It should be noted that the number of drift dives identified along the trips of the seals, especially for the adult animals, was low. In addition, some of the drift dives identified could be misclassified, because of the low number of inflection points reported for the compressed dives (for instance V dives could have been classified as drift dives). This problem was minimized in this study by integrating the probability of being a drift dive into the weighted beta splines; however, the results for this method should be interpreted with caution.

Each of the methods used in this study to identify hooded seal foraging areas had pros and cons. This is to be expected given that each is based on different assumptions. FPT is based on tracking data but does not take into account the vertical dimension of foraging, while the vertical transit rates and the change in drift rates methods are based on diving data without including horizontal aspects of spatial behavior. Taking the vertical dimension into account is important for deep diving predators like hooded seals, though it is likely less important for identifying foraging areas of more modest divers. The change in drift rate method clearly is the best method to infer fattening (foraging success), but changes in drift rates are hard to identify because relatively few drift dives are performed, so the two others methods have broader spatial coverage. In addition, foraging is not just fattening, maintenance is vital, which means that animal can forage successfully without showing an improvement in the body condition.

### Habitat preferences

Habitat assessment based on the search component of foraging, using either transmitted positions or the transmitted diving records, both highlighted the importance of bathymetry and SST to defining foraging areas. The similarities between what the tracks themselves suggested vs what diving behavior suggested in terms of hooded seal foraging habitat preferences in the NE population was reassuring; similar conditions were identified across a wide geographical area as being favorable for foraging. However, it is notable that vertical transit rates did not vary much with bathymetry, for any age/sex class, or season, while FPT did vary as a function of the bathymetry. FPTs revealed that adult males, adult females and pups focused their time in areas with water depths shallower than 500 m, 1,000 m and 2,000 m, respectively, whatever the season. This result supports earlier findings from both the NE and the NW hooded seals populations showing that even if this species is generally found in deep offshore waters, they prefer relatively shallow areas such as continental shelf breaks, submarine ridges or sea mounts when they are in search of food [39, 43, 92]. Such areas are generally zones of upwelling that have high biological productivity. However, in contrast to the NW population, which generally searched for food in relatively cold water [43–44], the seals in the present study focused their foraging effort in areas with SSTs of 4°C and higher in all seasons (4–8°C for pups and 8°C for adults). To some degree, the differences in SSTs preferences exhibited by the two stocks reflects the different oceanographic regimes occupied by these two populations. The East Greenland Current (EGC) transports ice and cold, low-salinity surface waters from the Arctic Ocean around Cape Farewell westward and forms the West Greenland Current and the Labrador Current, which are both cold-water systems that cover most of the distributional area of the NW Atlantic hooded seal population. In contrast, the North Atlantic Current, originating from the Gulf of Mexico, carries warm, salty surface waters northwards into the main distributional areas of the NE Atlantic hooded seal population.

The results from analyses of changes in drift rates produced somewhat different results compared with the other two methods, despite the identification of some common favorable foraging areas. Areas with positive change in drift rates were sometimes characterized by the same environmental variables as when using the FTP or vertical transit rate methods (i.e. shallow water depth and high SST). However, the drift rate method also identified areas with deeper and colder waters as important for improving body condition. This apparent contrast could be explained by the fact that the animals sometimes search without being very successful in catching prey, or alternatively that the animals might sometimes be very efficient in catching prey without much search effort. However, as discussed previously, the results from the drift dive analyses should be interpreted with some caution due to the low total number of drift dives identified.

All three methods support the fact that both bathymetry and SST are important variables for habitat selection for all age/sex classes of hooded seals. It is nevertheless important to note that these variables may be correlated with other habitat variables not included in the present study (such as chlorophyll, salinity and temperature at depth) rather than being habitat characteristics that directly determine the behavior and success of the animals.

The differences in habitat selection between the different age/sex classes, and also the differences in dive depths, as well as in the ratio between dive depth and bathymetry in the areas defined as being important for foraging, in combination suggest that the different age/sex classes are targeting different types of prey. The fact that pups dove significantly shallower than the adults in relative deeper areas could be due to physiological and behavioral constraints the pups face. While adults target some benthic prey, pups consume more pelagic fish than the adults, at least in the NW population [93]. Adult males and adult females explored areas with different bathymetry and explored different parts of the water column; with males foraging close to the bottom in shallow water areas, while females foraged relatively shallowly in deeper water areas. Potential prey species for the males that dove close to the bottom could include species such as Greenland halibut (*Reinhardtius hippoglossoides)*, redfish (*Sebastes* sp.), squid (*Gonatus fabricii)* or Atlantic cod (*Gadus morhua)*, that have all been identified previously in hooded seal stomachs [34, 94–96]. The females are obviously foraging on some type of pelagic prey, which could include species like polar cod (*Boreogadus saida*), capelin (*Mallotus villosus*) or herring, as well as Atlantic cod when this species is feeding up in the water column [34, 94–96].

### Conclusions

The present study used three different foraging indexes in combination, to identify and characterize important foraging areas for hooded seals in the NE Atlantic. All three methods highlighted some of the same geographic areas as being important foraging zones. However, they also suggested some important differences. When interpreted together, the differences provide important insights into the foraging ecology of hooded seals. For instance, the transit rate index indicated that the seals are likely foraging opportunistically along their entire path, without displaying ARS every time that they forage. If they do sometimes forage in a less concentrated manner, this energy intake may not result in detectible changes in drift rates. Even in areas where the seals do increase their search effort, they do not necessarily exhibit positive changes in drift rate, indicating that foraging success in some such areas might be low. Use of multiple indexes provided robust support for common findings, while also highlighting finer-scale patterns in the data that would have been missed if only a single method has been employed.

This study supports earlier findings that (i) adult male and adult female hooded seals from the NE population overlap spatially when dispersed at sea and that (ii) pup foraging is vertically segregated from that of adults. However, this study additionally showed that areas where adult seals forage show differences according to sex in terms of bathymetry; males and females feed in different parts of the water column. Females forage relatively shallowly in deep water areas, while males forage close to the bottom in more shallow areas. In addition, this study demonstrated that all NE Atlantic hooded seals selected areas with high SST when they were foraging, which differs from NW hooded seals.

## Supporting information

S1 Fig Trip identifications by individual. P, M and F represent pups, adult males and adult females, respectively. The IDs of individuals are detailed in parentheses. Brown represents the post-breeding season and orange represents the post-molting season. The horizontal line represents the 250 km threshold used for trip identification.
(TIF)

S2 Fig Trips made by male “2007–3” during the post-breeding and the post-molting season. Each color represents a trip.
(TIF)

S3 Fig Drift rates along 19 trips included in the drift dive analysis. P, M and F represent pups, adult males and adult females, respectively. The IDs of individuals are detailed in parentheses. Each dot corresponds to the drift rate of a drift dive. Red lines correspond to the constrained beta splines used to predict the daily drift rates along each trip. The size of the dots corresponds to the weight included into the splines, combining the probability of being a drift dive and the mean depth at which the descent ended and ascent began.
(TIF)
