# Supplementary figures and images for: Hooded seal Cystophora cristata foraging areas in the Northeast Atlantic Ocean—Investigated using three complementary methods
Source: PLoS One. 2017 Dec 6;12(12):e0187889. doi: 10.1371/journal.pone.0187889 (PMC5718402; doi:10.1371/journal.pone.0187889)

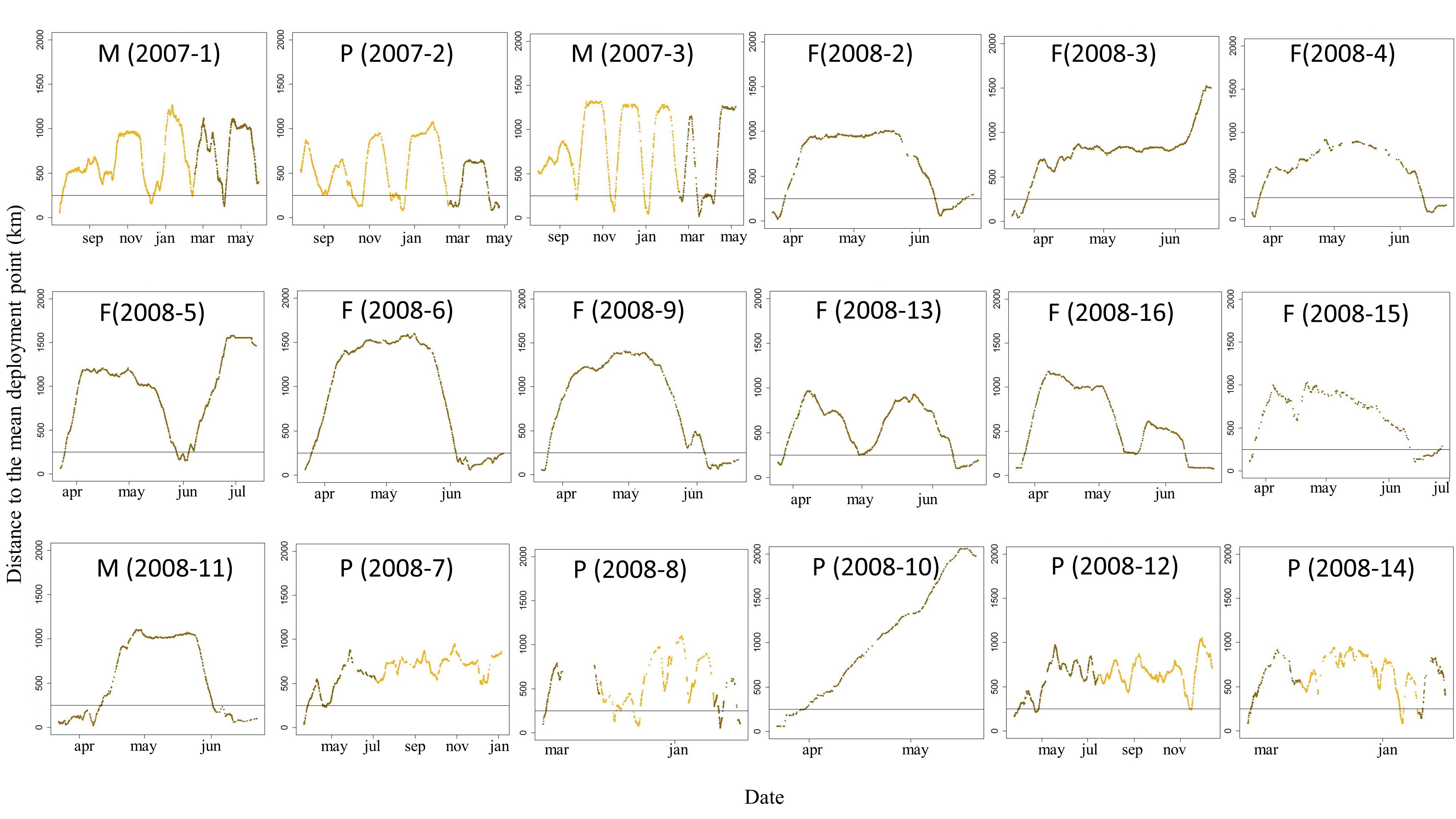

Supplement: S1 Fig — P, M and F represent pups, adult males and adult females, respectively. The IDs of individuals are detailed in parentheses. Brown represents the post-breeding season and orange represents the post-molting season. The horizontal line represents the 250 km threshold used for trip identification. (TIF) [file pone.0187889.s001.tif]

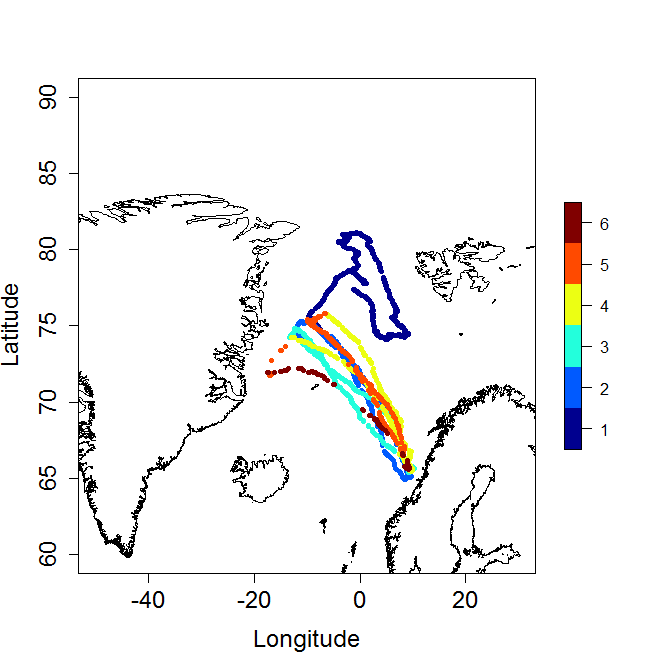

Supplement: S2 Fig — Each color represents a trip. (TIF) [file pone.0187889.s002.tif]

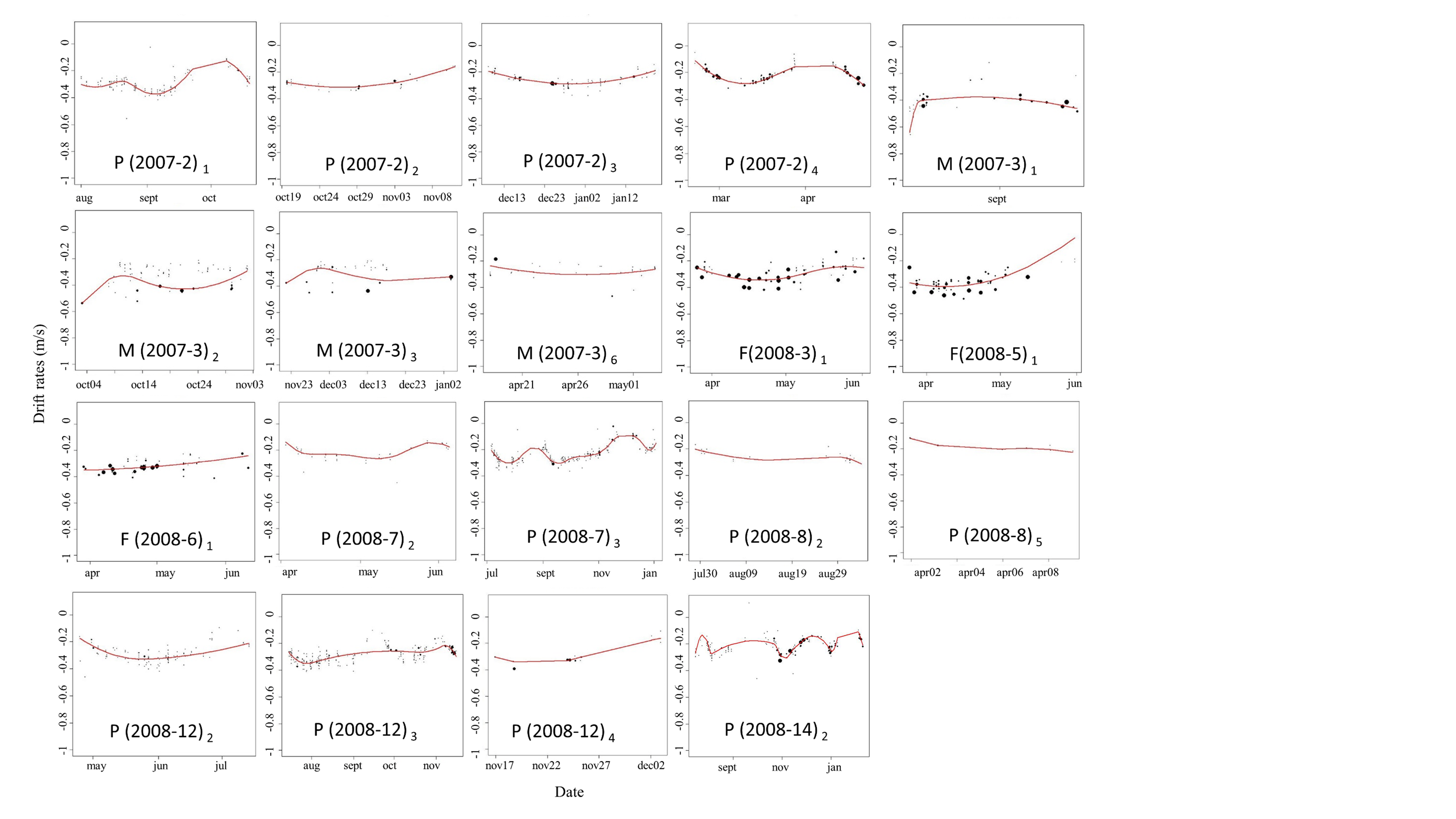

Supplement: S3 Fig — P, M and F represent pups, adult males and adult females, respectively. The IDs of individuals are detailed in parentheses. Each dot corresponds to the drift rate of a drift dive. Red lines correspond to the constrained beta splines used to predict the daily drift rates along each trip. The size of the dots corresponds to the weight included into the splines, combining the probability of being a drift dive and the mean depth at which the descent ended and ascent began. (TIF) [file pone.0187889.s003.tif]
